# Incident Diabetes in Women With Patterns of Gestational Diabetes Occurrences Across 2 Pregnancies

**DOI:** 10.1001/jamanetworkopen.2024.10279

**Published:** 2024-05-09

**Authors:** Joseph Mussa, Elham Rahme, Mourad Dahhou, Meranda Nakhla, Kaberi Dasgupta

**Affiliations:** 1 Department of Medicine, McGill University, Montreal, Quebec, Canada; 2 Centre for Outcomes Research and Evaluation (CORE), Research Institute of the McGill University Health Centre (RI-MUHC), Montreal, Quebec, Canada; 3 Department of Pediatrics, McGill University, Montreal, Quebec, Canada

## Abstract

**Importance:**

Gestational diabetes is a type 2 diabetes risk indicator, and recurrence further augments risk. In women with a single occurrence across 2 pregnancies, it is unclear whether first- vs second-pregnancy gestational diabetes differ in terms of risk.

**Objective:**

To compare the hazards of incident diabetes among those with gestational diabetes in the first, in the second, and in both pregnancies with women without gestational diabetes in either.

**Design, Setting, and Participants:**

This was a retrospective cohort study with cohort inception from April 1, 1990, to December 31, 2012. Follow-up was April 1, 1990, to April 1, 2019. Participants were mothers with 2 singleton deliveries between April 1, 1990, and December 31, 2012, without diabetes before or between pregnancies, who were listed in public health care insurance administrative databases and birth, stillbirth, and death registries in Quebec, Canada. Data were analyzed from July to December 2023.

**Exposure:**

Gestational diabetes occurrence(s) across 2 pregnancies.

**Main outcomes and measures:**

Incident diabetes from the second delivery until a third pregnancy, death, or the end of the follow-up period, whichever occurred first.

**Results:**

The 431 980 women with 2 singleton deliveries studied had a mean (SD) age of 30.1 (4.5) years at second delivery, with a mean (SD) of 2.8 (1.5) years elapsed between deliveries; 373 415 (86.4%) were of European background, and 78 770 (18.2%) were at the highest quintile of material deprivation. Overall, 10 920 women (2.5%) had gestational diabetes in their first pregnancy, 16 145 (3.7%) in their second, and 8255 (1.9%) in both (12 205 incident diabetes events; median [IQR] follow-up 11.5 [5.3-19.4] years). First pregnancy–only gestational diabetes increased hazards 4.35-fold (95% CI, 4.06-4.67), second pregnancy–only increased hazards 7.68-fold (95% CI, 7.31-8.07), and gestational diabetes in both pregnancies increased hazards 15.8-fold (95% CI, 15.0-16.6). Compared with first pregnancy–only gestational diabetes, second pregnancy–only gestational diabetes increased hazards by 76% (95% CI, 1.63-1.91), while gestational diabetes in both pregnancies increased it 3.63-fold (95% CI, 3.36-3.93).

**Conclusions and relevance:**

In this retrospective cohort study of nearly half a million women with 2 singleton pregnancies, both the number and ordinal pregnancy of any gestational diabetes occurrence increased diabetes risk. These considerations offer greater nuance than an ever or never gestational diabetes dichotomy.

## Key Points

**Question:**

Across 2 pregnancies, are the order and number of gestational diabetes occurrences linked to maternal diabetes risk in the years after second pregnancy?

**Findings:**

In this cohort study of 431 980 women, those with a first occurrence of gestational diabetes in a second pregnancy had a 76% higher risk for diabetes development than women who had gestational diabetes in the first pregnancy but not in the second, a statistically significant difference. The highest risk was in women with gestational diabetes in both pregnancies.

**Meaning:**

These findings suggest that considering gestational diabetes history in each pregnancy results in more accurate diabetes risk estimation than a simple yes/no dichotomy of past gestational diabetes occurrence.

## Teaser

This cohort study evaluated the hazards of incident diabetes among women in Canada with gestational diabetes in their first, second, and in both the first and second pregnancies.

## Introduction

Gestational diabetes (GD) affects 14% of pregnancies globally.^1^ A recent meta-analysis^2^ estimates its occurrence is associated with a 10-fold risk increase for type 2 diabetes. Whether risks vary with the order of GD occurrences is not well-studied. We hypothesized that new GD occurrence in a second pregnancy implies transition to a higher risk profile, while a single occurrence in a first pregnancy implies the converse.

One challenge is that GD is conditional on pregnancy (ie, cannot occur without pregnancy) and the number of pregnancies itself is associated with type 2 diabetes risk. The lowest risk occurs in those with 1 pregnancy.^3^ Two previous studies^4,5^ tried to improve comparability among participants by requiring that all have at least 2 pregnancies. Both reported a 2.4-fold increase in hazards with GD recurrence compared with its absence in the second pregnancy. They did not include women without any GD or women with a new occurrence of GD in a second pregnancy.

In the longer term, first-pregnancy GD may motivate some to adopt behaviors demonstrated to reduce diabetes risk,^6,7^ lowering GD recurrence rates and type 2 diabetes development. In contrast, some women without GD in the first pregnancy may enter a higher risk trajectory, related to excess gestational weight gain,^8^ postpartum weight retention,^9^ weight gain between pregnancies,^10,11^ and motherhood demands impeding healthy eating and physical activity.^12^ The delineation of differences in future incident type 2 diabetes risk between GD occurrence in a first pregnancy compared with new occurrence in a second could allow further personalization of approaches to type 2 diabetes prevention.^13^ We therefore examined patterns of GD absence, occurrence, and recurrence across 2 pregnancies and their associations with diabetes.

## Methods

The McGill University Health Centre’s research ethics board and Quebec Access to Information Commission approved the protocol. These bodies waived informed consent because the study involved deidentified data, analyses at the Quebec Statistical Institute’s secure data centers, and rounded frequencies to multiples of 5. This cohort study followed the Strengthening the Reporting of Observational Studies in Epidemiology (STROBE) reporting guidelines.

### Design and Data Sources

We conducted a retrospective cohort study in Quebec, Canada. We examined health administrative databases of the public health insurance plan linked to birth, stillbirth, and death registries by the Quebec Statistical Institute (probabilistic linkage). We obtained mothers’ residential territory and month and year of birth from the public health insurance registry. The Physician Services Claims and Hospitalization Discharge Databases include diagnostic codes (eTable 1 in Supplement 1) and hospitalization dates; we used these to define outcomes, exposures, and other variables alongside data from birth and stillbirth registries (offspring birthdates, gestational age at birth, birthweight, parental country of birth and first language, and years of maternal education). We also had access to the mothers’ Institut national de santé publique du Québec (INSPQ) material and social deprivation index, derived from the 6-digit postal code in the public health registry.^14^The Institut national de santé publique du Québec (INSPQ) material and social deprivation index is computed from small-area census data. Specifically, the material indices are derived from average income, proportions without high school diploma, and employment to population ratio among those 15 years and older. The social indices are derived from the proportion of the population who are single-parent families, aged 15 years and older living alone, and aged 15 years and older who are separated, divorced, or widowed. To assign the INSPQ index for each woman, we first checked availability of this variable in the index year (year of second delivery).

### Study Population

We considered women with 2 or more consecutive singleton deliveries between April 1, 1990, and December 31, 2012, who were alive at 12 weeks following the second delivery (index date) (eFigure 1 in Supplement 1). We excluded mothers with missing offspring gestational age (required to distinguish diabetes from GD),^15^ and those with diabetes or hypertension before or between pregnancies. We applied the validated Canadian Chronic Disease Surveillance System (CCDSS) diabetes^16,17^ and hypertension^18^ definitions of 2 outpatient or 1 hospitalization diagnostic code(s) to (1) the 2-year period before 20 weeks’ gestation in first pregnancy and (2) the period from 12 weeks after the first delivery until 20 weeks’ gestation in the second pregnancy. All required an opportunity to develop GD for both of the pregnancies considered, thus gestation 20 or more weeks was required. In the primary analysis, we required the same partner for each offspring to minimize heterogeneity of within-household factors.^19,20,21^ This resulted in the removal of stillbirths, for whom paternal data were unavailable; in a sensitivity analysis, we removed the paternal data requirement. Lastly, we excluded those with 2 outpatient visits or 1 hospitalization for cardiovascular disease, the most common diabetes consequence, before the index date.

### Exposure

We adapted a validated health administrative database GD definition^22^ that applies diabetes and GD diagnostic codes to a pregnancy-specific period. We started this period at 20 weeks’ gestation instead of the 120-day predelivery date used in the validation study, as the information we had on gestational age allowed us to conform with clinical definitions, which considers type 2 diabetes before 20 weeks’ gestation as preexisting.^15^ We extended the period beyond delivery to 12 weeks post partum, as screening for type 2 diabetes after pregnancy is generally advised by this time.^23,24^ We required 2 outpatient and/or 1 hospitalization code to maximize specificity (99.5%) and maintain sensitivity (94.1%), as recommended in the validation study.^22^ Our 4 mutually exclusive exposure categories were absence of GD, its presence in only first pregnancy, in only the second, and in both.

### Outcome

Our primary outcome was incident diabetes, using the previously described CCDSS definition.^16,17^ These diagnostic codes cannot differentiate between type 1 and type 2 diabetes, but because 95% of incident diabetes among adults is type 2 diabetes, they primarily capture incident type 2 diabetes. Follow-up was until the first of incident diabetes, the 120-day time point before a third delivery (we did not have gestational age data for any third pregnancy), death, or the end of the study period (April 1, 2019).

### Covariates

For both the first and second pregnancies, we considered other pregnancy and offspring-related factors associated with type 2 diabetes development, specifically, gestational hypertension (with or without preeclampsia), preterm delivery (<37 weeks), and small-for-gestational-age (SGA) and large-for-gestational-age (LGA) status.^25,26^ For gestational hypertension, we applied the CCDSS hypertension definition to the same pregnancy periods for which we defined GD, and we also considered diagnostic codes for gestational hypertension and preeclampsia.

We considered time between deliveries, comorbid conditions, maternal age at index date, deprivation level (see Table 1 footnotes),^14^ preexisting paternal diabetes and hypertension (validated CCDSS definitions^16,17^ applied from 2 years before 20 weeks’ gestation in the first pregnancy to 12 weeks following the second delivery), and ethnocultural background (African/Caribbean [if born in West/South/East/Central Africa or first language was of Caribbean or African descent], Arabic [if born in the Arab league or first language was of Arabic or other North African or South-West Asian descent], Asian [if born in West/East/Central/South/Southeast/Pacific Asia or first language descends from these regions], European [if born in North America, South America, Central America, Mexico, East/South/Southern/West Europe, or Australia and first language was English, French, or other European language], and other [if first language was of Indigenous descent) based on participant-reported place of birth and primary language recorded on the mandatory birth declaration and incorporated into the birth registry. Ethnocultural background was assessed in this study because those with background other than European have a higher baseline risk for diabetes.

**Table 1.** Baseline Covariates, Stratified by Gestational Diabetes (GD) Occurrence(s) in Each Respective Pregnancy

Covariate	Patients, No. (%)^a^			
	No GD (n = 396 660)	GD in first pregnancy (n = 10 920)	GD in second pregnancy (n = 16 145)	GD in both pregnancies (n = 8255)
Prior history of gestational hypertension in either or both pregnancies^b^	29 550 (7.5)	1275 (11.7)	2160 (13.4)	1160 (14.1)
Age of mother at second delivery, mean (SD), y^c^	30.0 (4.5)	30.9 (4.6)	31.6 (4.7)	32.0 (4.7)
Time between deliveries, y				
<2	124 800 (31.5)	3525 (32.3)	4055 (25.1)	2530 (30.6)
2 to <2.5	81 610 (20.6)	2250 (20.6)	2705 (16.8)	1545 (18.7)
2.5 to <3.5	103 195 (26.0)	2710 (24.8)	4010 (24.8)	2090 (25.3)
≥3.5	87 050 (21.9)	2435 (22.3)	5380 (33.3)	2090 (25.3)
Material deprivation index, quintiles^d^				
1	81 265 (20.5)	2005 (18.4)	2900 (18.0)	1475 (17.9)
2	83 975 (21.2)	2285 (20.9)	3250 (20.1)	1630 (19.7)
3	78 870 (19.9)	2135 (19.6)	3095 (19.2)	1560 (18.9)
4	74 525 (18.8)	2125 (19.5)	3155 (19.5)	1625 (19.7)
5	71 280 (18.0)	2195 (20.1)	3455 (21.4)	1840 (22.3)
Social deprivation index, quintiles^d^				
1	88 545 (22.3)	2305 (21.1)	3145 (19.5)	1760 (21.3)
2	85 620 (21.6)	2250 (20.6)	3255 (20.2)	1615 (19.6)
3	81 225 (20.5)	2200 (20.1)	3325 (20.6)	1605 (19.4)
4	72 980 (18.4)	2110 (19.3)	3155 (19.5)	1685 (20.4)
5	61 545 (15.5)	1885 (17.3)	2975 (18.4)	1465 (17.7)
Background^e^				
America, Australia, or Europe	346 230 (87.3)	8865 (81.2)	12 280 (76.1)	6040 (73.2)
Africa or Caribbean	7565 (1.9)	260 (2.4)	465 (2.9)	260 (3.2)
Arab-speaking regions	14 840 (3.7)	600 (5.5)	1210 (7.5)	665 (8.1)
Asia	12 040 (3.0)	640 (5.9)	1190 (7.4)	745 (9.0)
Other	15 980 (4.0)	555 (5.1)	1005 (6.2)	545 (6.6)
Comorbid conditions				
Mood disorders and alcohol or drug dependence	16 315 (4.1)	440 (4.0)	850 (5.3)	405 (4.9)
Thyroid disorder	13 185 (3.3)	450 (4.1)	895 (5.5)	480 (5.8)
Arthritis	8265 (2.1)	270 (2.5)	435 (2.7)	210 (2.5)
Asthma or COPD	7735 (2.0)	250 (2.3)	425 (2.6)	240 (2.9)
Small for gestational age^f^				
Neither pregnancy	338 405 (85.3)	9465 (86.7)	14 120 (87.5)	7235 (87.6)
First pregnancy only	24 225 (6.1)	630 (5.8)	815 (5.1)	445 (5.4)
Second pregnancy only	24 240 (6.1)	580 (5.3)	890 (5.5)	435 (5.3)
Both pregnancies	9650 (2.4)	240 (2.2)	320 (2.0)	135 (1.6)
Large for gestational age^f^				
Neither pregnancy	339 910 (85.7)	8735 (80.0)	12 635 (78.3)	6355 (77.0)
First pregnancy only	23 290 (5.9)	845 (7.7)	1315 (8.1)	710 (8.6)
Second pregnancy only	23 145 (5.8)	865 (7.9)	1370 (8.5)	690 (8.4)
Both pregnancies	10 175 (2.6)	470 (4.3)	825 (5.1)	495 (6.0)
Preterm birth				
Neither pregnancy	361 365 (91.1)	9725 (89.1)	14 040 (87.0)	7160 (86.7)
First pregnancy only	18 155 (4.6)	595 (5.5)	1050 (6.5)	530 (6.4)
Second pregnancy only	13 050 (3.3)	450 (4.1)	720 (4.5)	410 (5.0)
Both pregnancies	4085 (1.0)	150 (1.4)	340 (2.1)	155 (1.9)
History of paternal diabetes^g^	3170 (0.8)	110 (1.0)	225 (1.4)	140 (1.7)
History of paternal hypertension^g^	8385 (2.1)	305 (2.8)	480 (3.0)	250 (3.0)

Abbreviaton: COPD, chronic obstructive pulmonary disease.

^a^ Values are randomly rounded up or down to a multiple of 5 (for patient confidentiality purposes). Therefore, column sums for each baseline characteristic may not equal the total number of women in each level of the exposure due to this random rounding process.

^b^ Gestational hypertension was collapsed as a binary variable (absent or present in either or both pregnancies) as a measure to ensure the proportional hazards assumption was met when tested. When gestational hypertension status was categorized into 4 levels, similar to the primary GD exposure, the assumption was violated. The capture period for this comorbidity was between 20 weeks’ gestation to 12 weeks post partum of each respective pregnancy.

^c^ Age was not categorized and instead kept as a continuous variable.

^d^ Range from 1 (least deprived) to 5 (most deprived). A total of 7335 women were missing an assigned deprivation score.

^e^ Ethnocultural background based on the mother’s region of birth and reported preferred language. Women were categorized as European if born in North America, South America, Central America, Mexico, East/South/Southern/West Europe, or Australia and first language was English, French, or other European language; African or Caribbean if born in West/South/East/Central Africa or first language was of Caribbean or African descent; Arabic if born in the Arab league or first language was of Arabic or other North African or Southwest Asian descent; Asian if born in West/East/Central/South/Southeast/Pacific Asia or first language descends from these regions; or Other (does not fit into any other category), or if first language was of Indigenous descent.

^f^ A total of 150 offspring were missing birthweight required to derive offspring size.

^g^ Prior history in the father was defined as 1 or more inpatient and/or 2 or more outpatient *International Statistical Classification of Diseases and Related Health Problems, Ninth* and* Tenth Revision* codes for any form of diabetes or hypertension, respectively, that occurred during the period from 2 years before 20 weeks’ gestation in their partner’s first pregnancy to 12 weeks post partum in relationship to the second pregnancy.

### Statistical Analyses

We computed baseline characteristics (counts and proportions for categorical variables and mean [SD] for continuous variables) and compared them across exposure groups (Pearson χ^2^ tests for proportions; 1-way analysis of variance for means, as applicable). We calculated type 2 diabetes incidence rates (IR). We assessed for interactions (*P* < .05 for interaction terms) and multicollinearity (Cramer V > 0.10) among exposures and covariates. We constructed Kaplan-Meier curves and compared these through log-rank testing. We evaluated proportional hazards assumptions (log-minus-log survival plots, Schoenfeld residuals) and performed some transformations to fulfill these (age as a spline variable and binary gestational hypertension category defined as presence in either or both pregnancies vs neither).

We constructed multivariable Cox proportional hazards models to compute hazard ratios (HR) for type 2 diabetes, first with GD absence in either pregnancy as the reference group. We then examined models with GD in only the first pregnancy as the reference group and finally with GD in only the second as the reference group. We retained covariates based on univariate association with type 2 diabetes where *P* ≤ .25, multivariable association (stepwise selection) where *P* ≤ .05, and reduced bayesian information criteria values with inclusion (see eMethods in Supplement 1 for omitted variables).

In a sensitivity analysis, we evaluated the change in associations when including calendar years of each pregnancy in our models to account for temporal trends in the screening and diagnosis of GD over the years.^27^ In another sensitivity analysis, we retained women with stillbirth deliveries and accounted for stillbirths in the model, along with miscarriages between pregnancies. In a third sensitivity analysis, we applied indirect adjustments for obesity and smoking status to our main results, using established methods.^28,29^ This bias analysis required external estimates for the HRs of obesity and of smoking with incident type 2 diabetes in women, which we respectively estimated as 3.90 (obesity vs no obesity)^30^ and 1.13 (smoking vs not smoking).^31^ This method also required external cohort data for obesity and smoking prevalence in groups of women corresponding to our main exposure categories. We used the Canadian Community Health Survey (Cycle 2.2) for this purpose^32^; 13% were in the obesity category and 24% smoked cigarettes. We applied the following formula for the indirect obesity adjustment:

HR_(corrected for obesity)_ = HR_(from our analysis)_ / HR_(related to obesity, from literature)_P_oe_ − P_e_ × P_o_

(see eMethods in Supplement 1, similar formula applied for smoking; P_oe_ = proportion within specific GD category who have obesity; P_e_ = proportion of those with specific GD category among all women with 2 consecutive singleton pregnancies; P_o_ = proportion with obesity among all women with 2 consecutive singleton pregnancies). We performed analyses with SAS version 9.4 (SAS Institute). Data were analyzed from July 2023 to December 2023.

## Results

**Figure 1.**
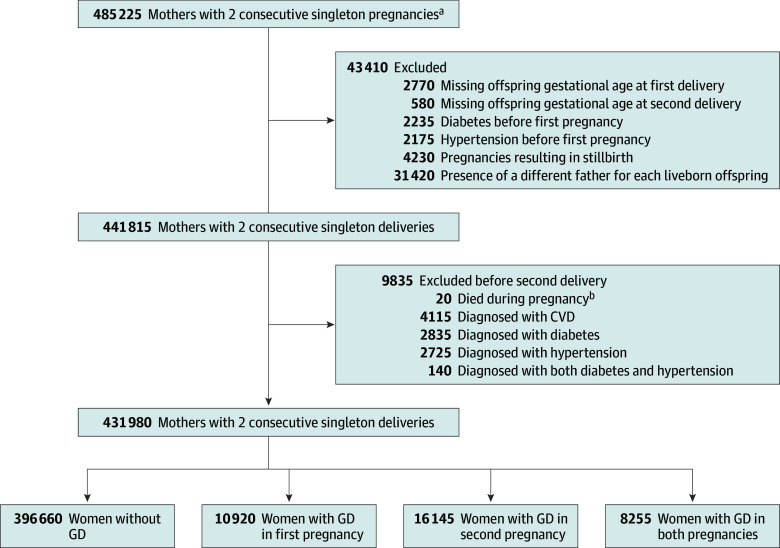
Cohort Construction ^a^Values are rounded either up or down to a multiple of 5 for patient confidentiality purposes. ^b^Fatal events occurring at any point between 20 weeks’ gestation in the second pregnancy and 12 weeks post partum. Five deaths were related to a fatal cardiovascular disease (CVD) event while the remaining 15 fatalities were related to obstetrical complications related to childbirth, major trauma, and suicide.

The 431 980 women analyzed (Figure 1) had a mean (SD) age of 30.1 (4.5) years, and a mean (SD) of 2.8 (1.5) years elapsed between deliveries. Overall, 8550 women were African or Caribbean, 17 315 were from Arab-speaking regions, 14 615 were Asian, 373 415 were of European background, 78 770 (18.2%) at the highest material deprivation level (INSPQ deprivation index: quintile 5), 62 605 (14.5%) had 1 or more SGA offspring, 64 195 (14.9%) had 1 or more LGA offspring, 35 290 (8.2%) had 1 or more preterm delivery, and 34 145 (7.9%) had 1 or more gestational hypertension occurrence. In terms of the main exposure, 10 920 (2.5%) had GD in only their first pregnancy, 16 145 (3.7%) had GD in only their second, and 8255 (1.9%) in both (eFigure 2 in Supplement 1). Those without GD in either pregnancy (396 660 participants) (Table 1) were younger with higher proportions of European background and lower proportions with deprivation, comorbid conditions, LGA offspring, preterm births, and partners with diabetes and hypertension.

### Associations of Main Exposure Groups With Incident Type 2 Diabetes

**Figure 2.**
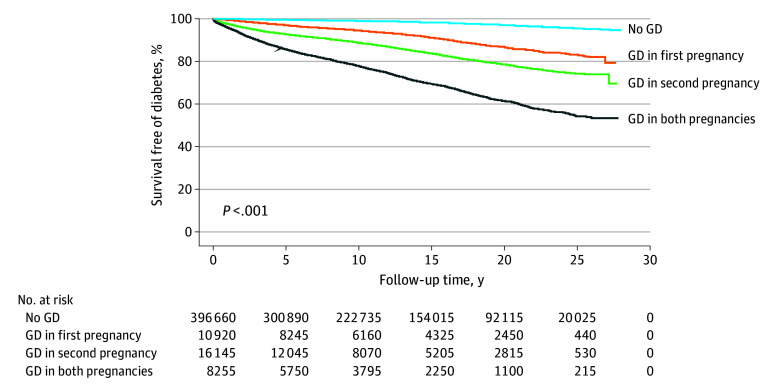
Kaplan-Meier Curves for Diabetes-Free Survival The log-rank test suggested significant differences in event-free survival across exposure groups. GD indicates gestational diabetes.

Over a median (IQR) of 11.5 (5.3-19.4) years (5 298 940 total person years), 12 205 mothers developed type 2 diabetes. The IRs per 1000 person-years rose across the no GD (IR, 1.4), GD in first pregnancy only (IR, 6.7), GD in second pregnancy only (IR, 12.4), and GD in both pregnancies (IR, 25.5) categories. Kaplan-Meier curves suggested significant differences in event-free survival across groups (Figure 2). The proportional hazards assumption applied. We did not detect interactions or multicollinearity.

**Figure 3.**
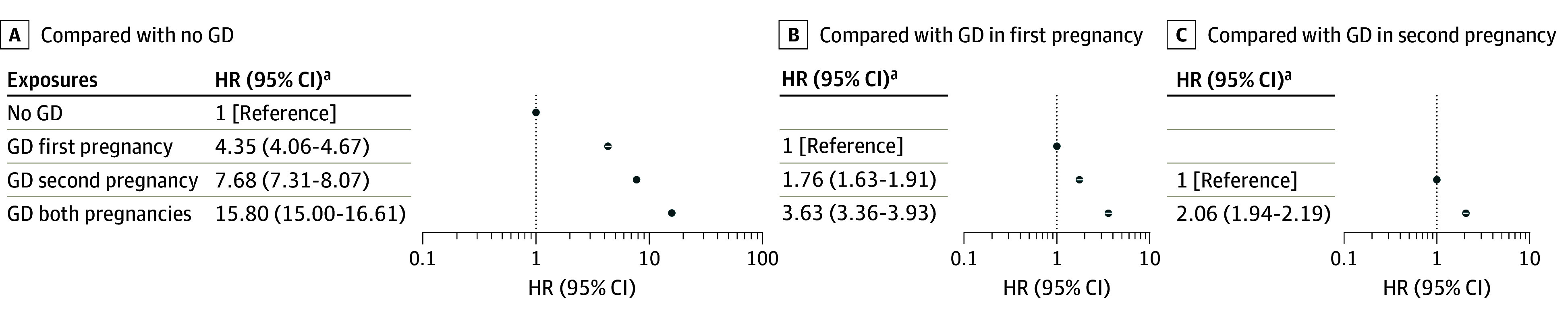
Associations of Incident Diabetes With Gestational Diabetes (GD) in First, Second, or Both Pregnancies, From Adjusted Multivariable Models The corresponding unadjusted hazard ratios (HRs) among women with no GD, GD in first pregnancy, GD in second pregnancy and GD in both pregnancies, in Panel A were 4.80 (95% CI, 4.48-5.14), 9.09 (95% CI, 8.67-9.53), and 19.05 (95% CI, 18.10-19.91) for GD in first pregnancy, GD in second pregnancy, and GD in both pregnancies, respectively. The corresponding unadjusted HRs among those in panel B were 1.89 (95% CI, 1.75-2.05) and 3.96 (95% CI, 3.66-4.28) for GD in second pregnancy and GD in both pregnancies, respectively. The corresponding unadjusted HR among those in panel C was 2.09 (95% CI, 1.97-2.22) for GD in both pregnancies.

In adjusted models, compared with those without GD, those with GD in first pregnancy had a 4.35-fold higher hazard for type 2 diabetes (95% CI, 4.06-4.67) (Figure 3A), those with GD in the second pregnancy had a 7.68-fold increase (95% CI, 7.31-8.07), and those with GD in both pregnancies demonstrated a 15.80-fold increase (95% CI, 15.00-16.61). Compared with those with GD in the first pregnancy, women with GD in the second had 76% higher hazards (95% CI, 1.63-1.91) (Figure 3B) and those with GD in both pregnancies had a 3.63-fold increase (95% CI, 3.36-3.93). Hazards were 2.06-fold higher among women with GD in both pregnancies (95% CI, 1.94-2.19) (Figure 3C) compared with those with GD in the second pregnancy.

### Sensitivity Analyses

Inclusion of calendar years for each pregnancy did not importantly alter HRs (eTable 2 in Supplement 1). In another sensitivity analysis including women with stillbirth pregnancies in our study cohort (435 685 participants; 12 415 events), stillbirths were associated with 19% increased hazards (HR, 1.19; 95% CI, 1.04-1.38), compared with women without stillbirth deliveries (eTable 3 in Supplement 1). Miscarriages between pregnancies were not conclusively associated with incident diabetes (HR, 1.03; 95% CI, 0.97-1.09). Furthermore, retaining stillbirths among the 2 pregnancies examined and considering miscarriages between pregnancies did not importantly alter the association between GD occurrences and incident diabetes.

Indirect adjustments for obesity (no GD: reference; GD in first pregnancy HR, 2.72; 95% CI, 2.46-2.83; GD in second pregnancy HR, 5.48; 95% CI, 5.22-5.76; GD in both pregnancies HR, 9.62; 95% CI, 9.15-10.10) (see eMethods in Supplement 1 for other comparisons) somewhat attenuated the HRs for the incident type 2 diabetes outcome. Indirect adjustments for smoking (no GD: reference; GD in first pregnancy HR, 4.23; 95% CI, 3.94-4.53; GD in second pregnancy HR, 7.44; 95% CI, 7.09-7.83; GD in both pregnancies HR, 15.50; 95% CI, 14.70-16.21) did not significantly alter the HRs.

### Other Associations Observed

Gestational hypertension in either or both pregnancies was associated with a 65% increase in hazards for diabetes development (Table 2). Compared with appropriate size for gestational age of both offspring, LGA was consistently associated with diabetes development, with a 60% increase in hazards whether it occurred in the first or second pregnancies, and a doubling when it occurred in both pregnancies or when LGA occurred in 1 pregnancy and SGA in the other. Preterm delivery in 1 or both pregnancies was associated with a 10% to 20% increase in hazards of diabetes compared with full-term delivery in both pregnancies. Deprivation levels were associated with a stepwise increase in hazards. All ethnocultural groups other than European had higher diabetes hazards compared with European women, and all comorbid conditions considered were associated with increased hazards. Paternal diabetes was associated with a 43% increase in hazards for maternal diabetes development.

**Table 2.** Association of Incident Diabetes With Covariates Included in the Final Model

Covariate^a^	HR (95% CI)	
	Unadjusted	Adjusted
Gestational hypertension affecting either pregnancy or both pregnancies^b^	2.14 (2.04-2.25)	1.65 (1.57-1.73)
Offspring indicators		
Offspring size		
AGA: both offspring	1 [Reference]	1 [Reference]
SGA: first offspring only	0.97 (0.90-1.05)	0.94 (0.87-1.02)
SGA: second offspring only	0.96 (0.89-1.04)	0.91 (0.84-1.00)
SGA: both offspring	1.00 (0.89-1.13)	0.97 (0.86-1.10)
LGA: first offspring only	1.82 (1.71-1.94)	1.60 (1.50-1.70)
LGA: second offspring only	1.86 (1.75-1.98)	1.60 (1.50-1.70)
LGA: both offspring	2.77 (2.57-2.98)	2.01 (1.86-2.17)
SGA: first offspring, LGA: second offspring	2.85 (2.02-4.01)	2.14 (1.51-3.02)
LGA: first offspring, SGA: second offspring	2.75 (1.95-3.87)	1.94 (1.38-2.73)
Gestational age of offspring at birth		
Term birth: both offspring	1 [Reference]	1 [Reference]
Preterm birth		
First offspring only	1.33 (1.23-1.44)	1.11 (1.03-1.20)
Second offspring only	1.50 (1.38-1.63)	1.19 (1.09-1.29)
Both offspring	1.71 (1.49-1.95)	1.21 (1.06-1.39)
Paternal indicators		
Prior history of paternal diabetes	2.09 (1.80-2.42)	1.43 (1.24-1.66)
Prior history of paternal hypertension	1.34 (1.20-1.49)	1.12 (1.00-1.25)
Maternal indicators		
Time between deliveries, y		
<2	1 [Reference]	1 [Reference]
2 to 2.5	0.85 (0.81-0.90)	0.90 (0.86-0.95)
2.5 to 3.5	0.82 (0.78-0.86)	0.84 (0.80-0.88)
≥3.5	1.06 (1.01-1.11)	0.94 (0.89-0.98)
Material deprivation index, quintiles^c^		
1 (Least deprived)	1 [Reference]	1 [Reference]
2	1.25 (1.17-1.32)	1.24 (1.17-1.32)
3	1.38 (1.30-1.47)	1.35 (1.27-1.43)
4	1.56 (1.47-1.66)	1.44 (1.36-1.53)
5 (Most deprived)	1.99 (1.87-2.10)	1.67 (1.58-1.78)
Social deprivation index, quintiles^c^		
1 (Least deprived)	1 [Reference]	1 [Reference]
2	1.08 (1.02-1.14)	1.08 (1.02-1.14)
3	1.14 (1.08-1.21)	1.10 (1.04-1.17)
4	1.32 (1.25-1.40)	1.16 (1.10-1.23)
5 (Most deprived)	1.53 (1.45-1.62)	1.26 (1.19-1.34)
Background^d^		
America, Australia, or Europe	1 [Reference]	1 [Reference]
Africa or Caribbean	2.68 (2.44-2.95)	1.90 (1.72-2.10)
Arab-speaking regions	2.24 (2.07-2.42)	1.60 (1.48-1.74)
Asia	2.50 (2.33-2.69)	1.62 (1.50-1.74)
Other	2.00 (1.86-2.15)	1.46 (1.35-1.58)
Comorbid conditions^e^		
Mood disorders and alcohol or drug dependence	1.50 (1.39-1.62)	1.40 (1.29-1.51)
Thyroid disorder	1.82 (1.68-1.98)	1.40 (1.29-1.53)
Arthritis	1.49 (1.35-1.65)	1.26 (1.14-1.40)
Asthma or COPD	1.95 (1.77-2.15)	1.67 (1.52-1.84)

Abbreviations: AGA, appropriate for gestational age; COPD, chronic obstructive pulmonary disease; HR, hazard ratio; LGA, large for gestational age; SGA, small for gestational age.

^a^ The Cox proportional hazards model adjusted for gestational diabetes occurrences across pregnancies, as well as each of the variables listed. Maternal age at second delivery was also included as a spline variable in the adjusted model; the corresponding unadjusted hazard ratio for each additional year in age was 1.03 (95% CI, 1.02-1.03).

^b^ Gestational hypertension was collapsed as a binary variable (absent or present in either or both pregnancies) as a measure to ensure the proportional hazards assumption was met when tested. When gestational hypertension status was categorized into 4 levels (similar to the primary GD exposure), the assumption was violated. The capture period for this comorbidity was between 20 weeks’ gestation to 12 weeks post partum of each respective pregnancy.

^c^ A total of 7335 women were missing a value for the material deprivation index.

^d^ Compared with women of European descent, those from other ethnic origins demonstrated increased hazards of developing type 2 diabetes during the follow-up period.

^e^ The reference group are women with the absence of each condition. Comorbid conditions were defined in accordance with the Chronic Disease Surveillance System’s definition of chronic disease, requiring 1 or more inpatient or 2 or more outpatient *International Classification of Diseases, Ninth *and* Tenth Revision* codes to be present within 2 years before the index date.

## Discussion

Among nearly half a million mothers with 2 consecutive singleton pregnancies, our analyses demonstrate that GD in only the second pregnancy was associated with higher hazards for type 2 diabetes development than GD in only the first pregnancy. The highest hazards are with GD occurrence in both pregnancies. Compared with women without GD in either pregnancy, there were 4.35-fold, 7.68-fold, and 15.80-fold greater hazards for type 2 diabetes with GD in the first, in the second, and in both pregnancies, respectively. Indirect adjustments for obesity somewhat attenuated these values to 2.72-fold, 5.48-fold, and 9.62-fold, respectively, but the magnitude remained high, and the differences persisted. Direct comparisons between GD groups were also conclusive. For example, compared with first pregnancy–only GD, second pregnancy–only GD increased hazards by 76%, while GD in both pregnancies increased hazards 3.63-fold.

We did not identify any prior study that compared GD in a first pregnancy with GD in a second among women with 2 pregnancies. Our specific estimate for the increase in hazards associated with GD recurrence (HR, 3.63; HR, 2.21 with indirect adjustment for obesity) compared with women with a GD occurrence in only a first pregnancy was similar to the greater than 2-fold increase in hazards reported in 2 previous studies^4,5^ that restricted analyses to women with at least 2 pregnancies. Other studies that examined GD recurrence had a higher degree of variability in numbers of pregnancies. One reported a 16% increase in hazards with GD recurrence^33^ while the other estimated a 2-fold increase.^34^ We identified a single study that examined hazards of type 2 diabetes after pregnancy in relationship to numbers of prior GD pregnancies (Sister Study).^35^ It differed in several other respects from ours. As such, its interpretation applies to an older group of women, several years beyond pregnancy. In the Sister Study, women with a history of 2 GD pregnancies experienced 6.2-fold higher hazards for type 2 diabetes in middle age compared with women with no GD pregnancies. The reference group included women without pregnancies. The investigators accounted for time since last GD pregnancy and self-reported weight. In our study, women with 2 pregnancies and GD in both had a 15.8-fold increase in hazards for type 2 diabetes development between their 30s and 40s, starting soon after their second pregnancy, compared with women without GD in either pregnancy. We indirectly adjusted for obesity and demonstrated that the association was attenuated to a 10-fold increase in hazards.

Our key discovery is that a single GD occurrence in a first pregnancy is associated with lower hazards for type 2 diabetes than a single GD occurrence in a second pregnancy. A subgroup analysis of the American Diabetes Prevention Program among women with a GD history showed that healthy diet-induced weight loss and higher physical activity levels could reduce type 2 diabetes risk.^6,7^ Women with a first GD pregnancy may be motivated to adopt behavioral changes that both prevent GD in a second pregnancy and lower hazards of incident type 2 diabetes development thereafter. Supporting this, a recent cohort study^11^ determined that among women without GD in their first pregnancy, weight loss between pregnancies was associated with reduced risk for new occurrence of GD in a subsequent pregnancy. In another study^10^ among women with excess weight and GD in a first pregnancy, weight loss between pregnancies lowered the risk for GD recurrence in a second pregnancy.

In our analyses, the women without GD in the first pregnancy who developed GD in the second may have gained excess weight in the first pregnancy and had difficulty losing it^9^ or gained weight between pregnancies.^10,11^ In an Australian investigation^11^ among women without GD in their first pregnancy, higher levels of weight gain between pregnancies were associated with stepwise increases in the risk of new occurrence of GD in second pregnancy. For many women, the additional responsibilities of motherhood^12^ may challenge efforts to engage in behaviors to enhance personal health. Furthermore, the metabolic stresses inherent to pregnancy may impair their β-cell function,^36^ making them more susceptible to developing GD in the second pregnancy, and ultimately to type 2 diabetes development.

Alongside health behaviors and physiological changes, our analyses reinforce the importance of social factors, including material and social deprivation and non-European background. The underpinnings of such associations likely stem from other related upstream characteristics, such as food insecurity,^37^ local environments not conducive to physical activity,^38,39^ and structural inequity.^40^ Partner diabetes was another risk indicator for maternal type 2 diabetes development in our analyses, with a 43% increase in hazards. This is consistent with our prior studies^41,42^ demonstrating increases in hazards for the development of diabetes in fathers whose partners had GD compared with those whose partners did not. Shared partner type 2 diabetes risk may be related to shared health behaviors, resources, social factors, and household environments.^20,43,44,45,46^ Assortative mating (similar demographics, attitudes, behaviors, and traits at the outset) may also play a role.^46,47,48,49^

### Strengths and Limitations

Our large sample size of nearly half a million women was possible through linkage of Quebec’s health administrative and vital statistics databases. Limitations to these data include lack of information on GD management, prepartum weight status, gestational weight gain, smoking status, and laboratory values. GD and weight excess are intimately associated. However, all of our models accounted for LGA in both the first and second pregnancies, a strong correlate of prepregnancy and gestational weight gain.^50,51^ Furthermore, we performed indirect adjustments for obesity using established methods.^28,29^ We could not corroborate *International Statistical Classification of Diseases and Related Health Problems, Ninth *and *Tenth Revision*–coded diagnoses of diabetes with laboratory data, but to mitigate for misclassification, we applied validated definitions of GD and diabetes.^16,17^ The diagnostic codes do not reliably distinguish between type 1 and type 2 diabetes, but given that 95% of diabetes onset among adults is type 2 diabetes, the majority of events captured with our codes are type 2 diabetes. We acknowledge that women with more than 1 GD occurrence may already be undergoing more frequent screening for diabetes than women with only 1 occurrence, perhaps partly accounting for their higher diabetes hazards. We also acknowledge potential misclassification of ethnocultural background, as second- and third-generation women and/or Indigenous women would have been classified as European if their first language was English, French, or another European language. Lastly, we did not examine women without pregnancies or women with a single pregnancy, but our focus was women with 2 consecutive deliveries; restriction to women with 2 or more deliveries overcame some methodological challenges, as discussed.

## Conclusions

Our retrospective cohort study suggests that among women with 2 consecutive singleton pregnancies, without diabetes before or between pregnancies, the absence of GD in a second pregnancy following GD in the first suggests that the mother is taking effective diabetes prevention measures. If confirmed, she should be encouraged to continue. New onset GD or recurrent GD in a second pregnancy, however, should inspire urgent action for prevention or adjustments to ongoing efforts. We also confirmed the importance of material deprivation and ethnocultural background in type 2 diabetes risk estimation, and we identified paternal diabetes as a factor associated with risk for type 2 diabetes development in mothers. Our results provide a personalized medicine–oriented pathway to diabetes risk estimation in women. This should be coupled with tailored prevention programs and equitable referral pathways to reduce the burden of type 2 diabetes and its complications.

## Notes

Supplement 1. **eTable 1. ***ICD* Codes **eTable 2.** Comparing Effect Estimates of the Diabetes Outcome, With and Without Inclusion of Years of Pregnancy, with Account for Temporal Trends in the Screening and Diagnosis of Gestational Diabetes **eTable 3. **Associations of Incident Diabetes With Gestational Diabetes Occurrences When Including Women With Stillbirth Pregnancies in Cohort
eMethods.
**eFigure 1. **Timeline of Applied Exclusions and Exposure Ascertainment Windows **eFigure 2.** Distribution of Gestational Diabetes Exposure Categories

Supplement 2.
Data Sharing Statement
