# Supplementary material for: Incident Diabetes in Women With Patterns of Gestational Diabetes Occurrences Across 2 Pregnancies
Source: JAMA Netw Open. 2024 May 9;7(5):e2410279. doi: 10.1001/jamanetworkopen.2024.10279 (PMC11082690; doi:10.1001/jamanetworkopen.2024.10279)
Supplement: Supplement 1. — eTable 1. ICD Codes eTable 2. Comparing Effect Estimates of the Diabetes Outcome, With and Without Inclusion of Years of Pregnancy, with Account for Temporal Trends in the Screening and Diagnosis of Gestational Diabetes eTable 3. Associations of Incident Diabetes With Gestational Diabetes Occurrences When Including Women With Stillbirth Pregnancies in Cohort eMethods. eFigure 1. Timeline of Applied Exclusions and Exposure Ascertainment Windows eFigure 2. Distribution of Gestational Diabetes Exposure Categories [file jamanetwopen-e2410279-s001.pdf]

Supplemental Online Content

Mussa J, Rahme E, Dahhou M, Nakhla M, Dasgupta K. Incident diabetes in women with patterns of gestational diabetes occurrences across 2 pregnancies. *JAMA Netw Open*. 2024;7(5):e2410279. doi:10.1001/jamanetworkopen.2024.10279

eTable 1. *ICD* Codes
eTable 2. Comparing Effect Estimates of the Diabetes Outcome, With and Without Inclusion of Years of Pregnancy, to Account for Temporal Trends in the Screening and Diagnosis of Gestational Diabetes
eTable 3. Associations of Incident Diabetes With Gestational Diabetes Occurrences When Including Women With Stillbirth Pregnancies in Cohort
eMethods
eFigure 1. Timeline of Applied Exclusions and Exposure Ascertainment Windows
eFigure 2. Distribution of Gestational Diabetes Exposure Categories

This supplemental material has been provided by the authors to give readers additional information about their work.

© 2024 Mussa J et al. *JAMA Network Open.*

eTable 1. ICD codes

Condition	ICD-9	ICD-10	Capture period
Prior cardiovascular disease or circulatory system disease conditions (mother; exclusion criteria)[a]	325, 410-415, 427-444, 451-453, 6396, 671, 673, 6740, 7943, 9971-9972, 2506	I20-I26, I46-I52, I60-I70, I73-I74, I79-I82, I86, I97, R9430-R9431, E105, E115, E145, G08, G45-G46, H34, O882, O994, T817	2 years prior to 12 weeks' postpartum of second delivery (index date) – *2 outpatient diagnoses, 1 inpatient diagnosis or 1 related surgical procedure (angioplasty, endarterectomy or coronary artery bypass surgery)*
Prior diabetes (mother; exclusion criteria)	250, 6480, 6488	E10-14, O245-O248	2 years prior to 20 weeks' gestation of first pregnancy (preexisting condition) or codes occurring between 12 weeks' postpartum of the first pregnancy and 20 weeks' gestation of the second pregnancy (condition developed during interpregnancy interval) – *2 outpatient diagnoses (within 2 years) or 1 inpatient diagnosis*
Prior hypertension (mother; exclusion criteria)	401-405, 642	I10-I13, I15, O10-O11, O13-O16	2 years prior to 20 weeks' gestation of first pregnancy (preexisting condition) or codes occurring between 12 weeks' postpartum of the first pregnancy and 20 weeks' gestation of the second pregnancy (condition developed during interpregnancy interval) – *2 outpatient diagnoses (within 2 years) or 1 inpatient diagnosis*
Prior diabetes (father; covariate)	250, 6480, 6488	E10-14, O245-O248	2 years prior to 20 weeks' gestation of first pregnancy to 12 weeks' postpartum of second pregnancy – *2 outpatient diagnoses (within 2 years) or 1 inpatient diagnosis*
Prior hypertension (father; covariate)	401-405, 642	I10-I13, I15, O10-O11, O13-O16	2 years prior to 20 weeks' gestation of first pregnancy to 12 weeks' postpartum of second pregnancy – *2 outpatient diagnoses (within 2 years) or 1 inpatient diagnosis*
Gestational diabetes	250, 6480, 6488	E10-14, O248	20 weeks' gestation of each respective pregnancy to 12 weeks' postpartum – *2 outpatient diagnoses or 1 inpatient diagnosis*
Gestational hypertension (collapsed as a binary variable)[b]	401-405, 642	I10-I13, I15, O10-O11, O13-O16	20 weeks' gestation of each respective pregnancy to 12 weeks' postpartum – *2 outpatient diagnoses or 1 inpatient diagnosis*
Diabetes outcome	250, 6480, 6488	E10-14, O245-O248	After 12 weeks' postpartum of the second delivery (index date) – *2 outpatient diagnoses (within 2 years) or 1 inpatient diagnosis*
Pregnancy after the index date (censor at 120 days prior)	630-676, 763, 767-768, 779<br><br>V22-V24, V27-V39	O00-O99, Z32-Z39, P95, P964, P968-P969	After 12 weeks' postpartum of the second delivery (index date) – *1 inpatient diagnosis*
Miscarriages between pregnancies	630-638	O03-O05	12 weeks' postpartum of the first delivery to 20 weeks' gestation of the second pregnancy - *1 outpatient or 1 inpatient diagnosis*
Mood disorders, alcohol or drug dependence	291-292, 295-305, 311<br>V11 V654	F10-F25, F30-F34, F38-F45, F48, F53, F99<br>R457 Z914 Z915<br>X65 Z714 Z864-Z865	2 years prior to 12 weeks' postpartum of the second delivery (index date) – *2 outpatient diagnoses or 1 inpatient diagnosis*
Thyroid disorders	240-246<br>0175, 1222, 2513, 6481, 7753, 7758, 7945, V770	E01-E07<br>A188, B673, E350, E890-E891, O905, P720-P722, R946, Z138, O9920	2 years prior to 12 weeks' postpartum of the second delivery (index date) – *2 outpatient diagnoses or 1 inpatient diagnosis*
Arthritis	274<br>6960, 710-721, 724	M05-M19, M32, M43, M46-M48, M53-M54, L405	2 years prior to 12 weeks' postpartum of the second delivery (index date) – *2 outpatient diagnoses or 1 inpatient diagnosis*
Asthma or COPD	491-493, 496, 5181-5182	J44-J45	2 years prior to 12 weeks' postpartum of the second delivery (index date) – *2 outpatient diagnoses or 1 inpatient diagnosis*

COPD = chronic obstructive pulmonary disease; CVD = cardiovascular disease; HIV = human immunodeficiency virus

© 2024 Mussa J et al. *JAMA Network Open.*

[a]The following ***procedure codes*** related to pacemaker implantation, angioplasty, endarterectomy or coronary artery bypass surgery were additionally used for exclusions: 00460, 00631, 00662, 04022, 04030, 04031, 04037, 04046, 04601-04608, 04610- 04612, 04661, 04662, 04665-04668, 04669, 04689, 04692-04699, 04701-04704, 04707-04709, 04710, 04713-04716, 04721-04723, 04725-04727, 04732-04737, 04740-04058, 09302, 20123, 20124, 20186, 20191, 20194, 20195, 20531, 20532, 20577-20583-20590.

© 2024 Mussa J et al. *JAMA Network Open.*

eTable 2. Comparing effect estimates of the diabetes outcome, with and without inclusion of years of pregnancy, to account for temporal trends in the screening and diagnosis of gestational diabetes

**Exposure**	**Excluding years of pregnancy in model**	**Including years of pregnancy in model**[a]	**Absolute Δ HR for incident diabetes**
No GD	Reference	Reference	
GD in first pregnancy	4.35 (4.06-4.67)	4.36 (4.07-4.68)	+0.01
GD in second pregnancy	7.68 (7.31-8.07)	7.64 (7.28-8.03)	-0.04
GD in both pregnancies	15.80 (15.00-16.61)	15.80 (15.10-16.72)	0.00
GD in first pregnancy	Reference	Reference	
GD in second pregnancy	1.76 (1.63-1.91)	1.75 (1.62-1.90)	-0.01
GD in both pregnancies	3.63 (3.36-3.93)	3.63 (3.36-3.93)	0.00
GD in second pregnancy	Reference	Reference	
GD in both pregnancies	2.06 (1.94-2.19)	2.07 (1.95-2.20)	+0.01

GD = gestational diabetes; HR = hazard ratio

[a] These analyses include all of the baseline characteristics shown in Table 2, in addition to calendar years for pregnancy #1 and pregnancy #2 (as two separate variables).

© 2024 Mussa J et al. *JAMA Network Open.*

eTable 3. Associations of incident diabetes with gestational diabetes occurrences when including women with stillbirth pregnancies in cohort (N=435,685)

	Variable	Adjusted hazard ratio (95% CI) from sensitivity analysis
Exposure	**Gestational diabetes occurrences**	
	No GD	*Reference*
	GD in first pregnancy	4.34 (4.05-4.65)
	GD in second pregnancy	7.65 (7.28-8.03)
	GD in both pregnancies	15.80 (15.00-16.61)
	**Gestational hypertension affecting either pregnancy or both pregnancies**	1.63 (1.55-1.72)
Offspring indicators	**Offspring size**	
	AGA: both offspring	*Reference*
	SGA: 1st offspring only	0.94 (0.87-1.02)
	SGA: 2nd offspring only	0.91 (0.84-1.00)
	SGA: both offspring	0.99 (0.88-1.11)
	LGA: 1st offspring only	1.61 (1.51-1.71)
	LGA: 2nd offspring only	1.60 (1.50-1.70)
	LGA: both offspring	2.01 (1.87-2.17)
	SGA: 1st offspring , LGA: 2nd offspring	2.13 (1.51-3.02)
	LGA: 1st offspring , SGA: 2nd offspring	1.94 (1.38-2.73)
	**Gestational age of offspring at birth**	
	Term birth: both offspring	*Reference*
	Preterm birth: 1st offspring only	1.09 (1.01-1.17)
	Preterm birth: 2nd offspring only	1.19 (1.09-1.29)
	Preterm birth: both offspring	1.19 (1.05-1.36)
Paternal	**Prior history of paternal diabetes**[a]	- -
	**Prior history of paternal hypertension**[a]	- -
Maternal indicators	**Age of mother at 2nd delivery, years**	
	Not applicable as a result of spline.	
	**Stillbirth delivery**[b]	
	Yes	1.19 (1.04-1.38)
	**Miscarriage between pregnancies**[c]	
	Yes	1.03 (0.97-1.09)
	**Time between deliveries, years**	
	<2	*Reference*
	2-2.5	0.90 (0.85-0.95)
	2.5-3.5	0.84 (0.80-0.88)

© 2024 Mussa J et al. *JAMA Network Open.*

	≥3.5	0.94 (0.90-0.98)
	**Material deprivation index, quintiles**	
	1 (least deprived)	*Reference*
	2	1.24 (1.17-1.32)
	3	1.34 (1.26-1.43)
	4	1.44 (1.36-1.53)
	5 (most deprived)	1.67 (1.57-1.77)
	**Social deprivation index, quintiles**	
	1 (least deprived)	*Reference*
	2	1.07 (1.02-1.14)
	3	1.10 (1.04-1.17)
	4	1.16 (1.10-1.23)
	5 (most deprived)	1.26 (1.19-1.33)
	**Background**	
	America, Australia or Europe	*Reference*
	Africa or Caribbean	1.89 (1.72-2.08)
	Arab-speaking regions	1.59 (1.47-1.72)
	Asia	1.63 (1.51-1.75)
	Other	1.46 (1.36-1.58)
	**Co-morbid conditions**	
	Mood disorders, alcohol or drug dependence	1.39 (1.29-1.50)
	Thyroid disorder	1.41 (1.30-1.53)
	Arthritis	1.26 (1.14-1.39)
	Asthma or COPD	1.67 (1.52-1.84)

[a] Prior history of paternal diabetes and hypertension were removed from the Cox proportional hazards model in this sensitivity analysis, as linked paternal information is unavailable for stillbirths.

[b] 3,705 women had a first and/or second pregnancy that resulted in a stillbirth.

[c] 51,360 women with miscarriage between pregnancies. We required at least one outpatient and hospitalization record of miscarriage/abortion (ICD-9: 630-638, ICD-10: O03-O05) occurring from 12 weeks postpartum of the first pregnancy and 20 weeks' gestation of the second pregnancy to define a miscarriage between pregnancies. This variable was removed from our primary analysis because it did not meet our criteria for variable inclusion (see Statistical Analysis).

© 2024 Mussa J et al. *JAMA Network Open.*

eMethods

Omitted variables from primary analysis (variable selection): Other variables that we considered but ultimately did not meet thresholds for inclusion in statistical models (see Statistical Analysis for inclusion criteria) were several paternal variables (age, ethnicity), parental co-habitation, years of maternal education, offspring congenital anomalies, offspring sex, history of cancer, history of HIV or chronic hepatitis, placental abruption and miscarriages between pregnancies.

Sensitivity analysis (adjusting for unmeasured confounders):

Shin et al., 2014 (1); Lash et al., 2014 (2)

$$HR_{(\text{corrected for smoking})} = \frac{HR_{(\text{from our analysis})}}{HR_{(\text{related to smoking, from literature})}^{\,P_{se}-P_eP_s}}$$

Notation

P_{se} = proportion within specific exposure category who smoke

P_e = proportion of those corresponding to specific exposure category among all women with two consecutive singleton pregnancies

P_s = proportion who smoke among all women with two consecutive singleton pregnancies

$HR_{(\text{related to smoking, from literature})}$ = 1.13 (3)
$HR_{(\text{related to obesity, from literature})}$ = 3.90 (4)

GD occurrences from CCHS-derived cohort				
	**No GD**	**GD in 1st pregnancy**	**GD in 2nd pregnancy**	**GD in both pregnancies**
P_{se}: Proportion of Smokers (%, N)	24.34 (266/1093)	25.00 (8/32)	27.03 (20/74)	18.75 (6/32)
P_{oe}: Proportion of Obese (%, N)*	12.35 (74/599)	35.00 (7/20)	25.64 (10/39)	36.84 (7/19)

*554 women missing BMI measures in CCHS, cycle 2.2

Methods for applying indirect adjustment: We performed a bias analysis that indirectly adjusted for obesity and smoking status using data from the Canadian Community Health Survey (CCHS) with established methods.[1,2] CCHS 2004-2005 incorporated direct measurements of height and weight and queried smoking status (never, occasional, and daily). These data were linked to hospital discharge diagnoses and mortality data. This sample is inherently designed by Statistics Canada to be a representative sample of the Canadian population; we applied specific inclusion criteria (e.g., limited to women aged 12-50 with at least two pregnancies recorded between 2004-2017; without prior diabetes, hypertension, or CVD at baseline) in an attempt to mimic the inclusion criteria applied to our primary cohort. This was performed to maximize subject comparability between both datasets. We classified the 1,231 women who met these inclusion criteria by GD status, and computed proportions smoking (occasional smoker and daily smoker vs. non-smoker) and/or with obesity at baseline. We then adjusted the hazard ratios (HRs) and confidence intervals (CIs) corresponding to the primary GD exposures in the present study, with these proportions. The methods for indirect adjustment for smoking and obesity (using data available in the CCHS), proposed by Shin and associates, were previously validated with CVD outcomes and are described in detail elsewhere.[1,2]Adjusting for unmeasured confounders

© 2024 Mussa J et al. *JAMA Network Open.*

using this method requires the model to adjust for one unmeasured variable at a time and for the unmeasured variable to be dichotomous.

Application to our findings: When the adjusted hazard ratios (shown in Figure 3) were additionally indirectly adjusted for obesity, the following hazard ratios were obtained for those in Panel A: 1.00 (reference), 2.72 (95%CI 2.46-2.83), 5.48 (95%CI 5.22-5.76) and 9.62 (95%CI 9.15-10.10), for women with no GD, GD in first pregnancy, GD in second pregnancy and GD in both pregnancies, respectively. Indirect adjustments for obesity resulted in the following hazard ratios for those in Panel B: 1.00 (reference), 1.26 (95%CI 1.17-1.37) and 2.21 (95%CI 2.05-2.39), respectively. The following results were obtained for this indirect adjustment among those in Panel C: 1.00 (reference) and 1.25 (95%CI 1.18-1.33).

When the adjusted hazard ratios were additionally indirectly adjusted for smoking, the following hazard ratios were obtained for those in Panel A: 1.00 (reference), 4.23 (95%CI 3.94-4.53), 7.44 (95%CI 7.09-7.83) and 15.50 (95%CI 14.70-16.21), for those with no GD, GD in first pregnancy, GD in second pregnancy and GD in both pregnancies, respectively. Indirect adjustments for smoking resulted in the following hazard ratios for those in Panel B: 1.00 (reference), 1.71 (95%CI 1.58-1.85) and 3.55 (95%CI 3.29-3.85), respectively. The following results were obtained for this indirect adjustment among those in Panel C: 1.00 (reference) and 2.01 (95%CI 1.89-2.13).

References

1. Shin HH, Cakmak S, Brion O, et al. Indirect adjustment for multiple missing variables applicable to environmental epidemiology. *Environ Res*. Oct 2014;134:482-7. doi:10.1016/j.envres.2014.05.016
2. Lash TL, Fox MP, MacLehose RF, Maldonado G, McCandless LC, Greenland S. Good practices for quantitative bias analysis. *Int J Epidemiol*. Dec 2014;43(6):1969-85. doi:10.1093/ije/dyu149
3. Spijkerman AM, van der AD, Nilsson PM, et al. Smoking and long-term risk of type 2 diabetes: the EPIC-InterAct study in European populations. *Diabetes Care*. Dec 2014;37(12):3164-71. doi:10.2337/dc14-1020
4. Hinnouho GM, Czernichow S, Dugravot A, et al. Metabolically healthy obesity and the risk of cardiovascular disease and type 2 diabetes: the Whitehall II cohort study. *Eur Heart J*. Mar 1 2015;36(9):551-9. doi:10.1093/eurheartj/ehu123

© 2024 Mussa J et al. *JAMA Network Open.*

eFigure 1. Timeline of applied exclusions and exposure ascertainment windows

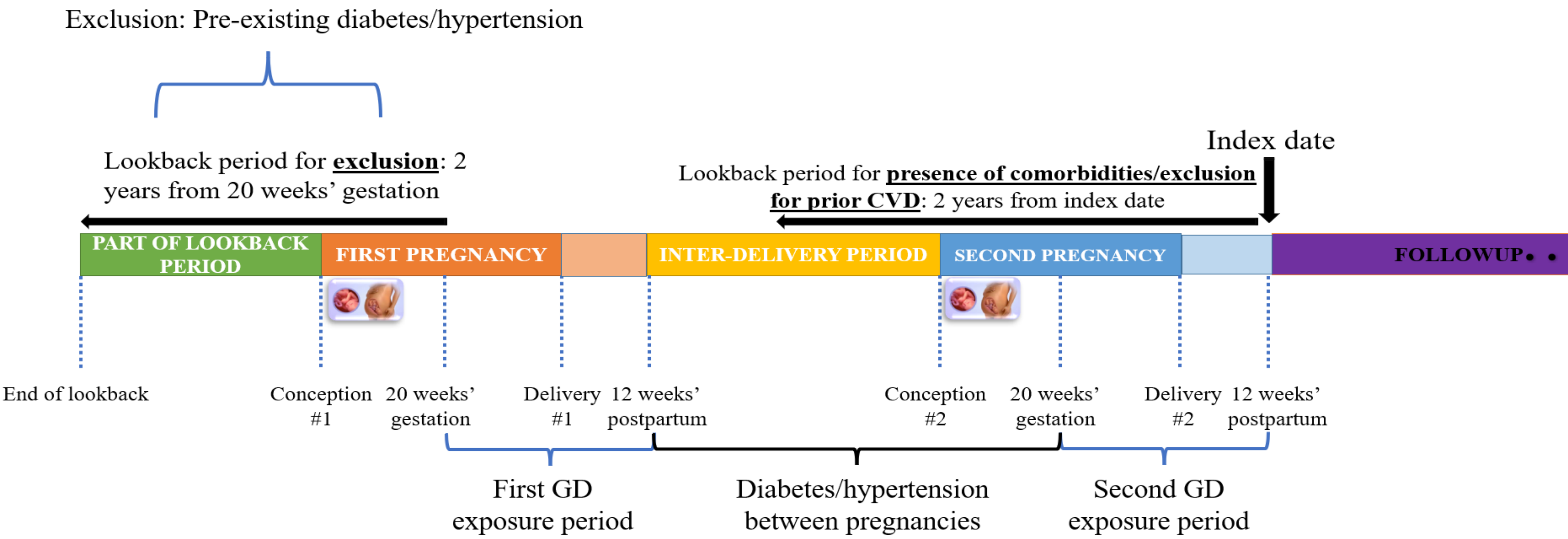

© 2024 Mussa J et al. *JAMA Network Open.*

eFigure 2. Distribution of gestational diabetes exposure categories

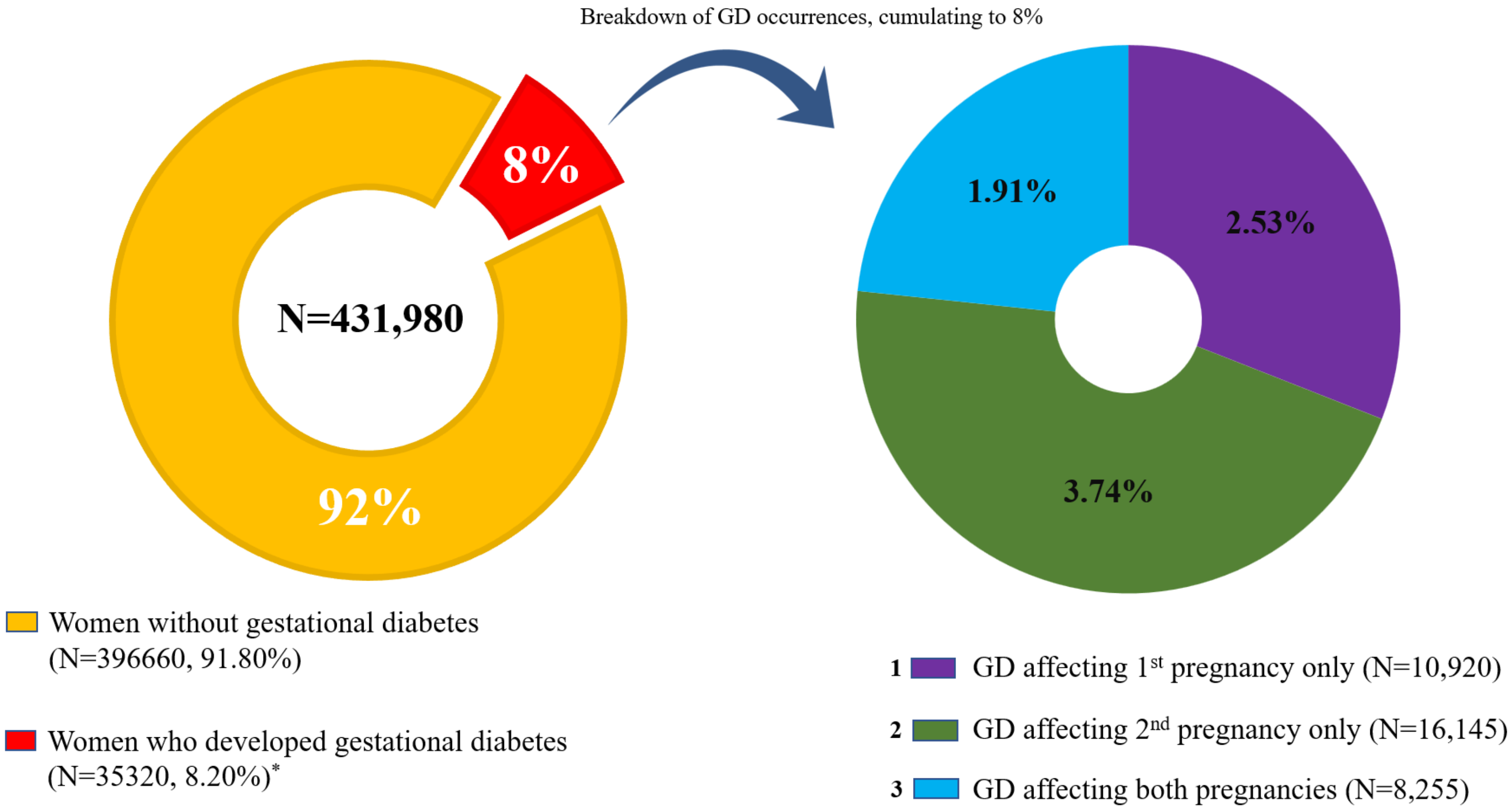

© 2024 Mussa J et al. *JAMA Network Open.*
