# Supplementary material for: Incident Diabetes in Women With Patterns of Gestational Diabetes Occurrences Across 2 Pregnancies
Source: JAMA Netw Open. 2024 May 9;7(5):e2410279. doi: 10.1001/jamanetworkopen.2024.10279 (PMC11082690; doi:10.1001/jamanetworkopen.2024.10279)
Supplement: Supplement 2. — Data Sharing Statement [file jamanetwopen-e2410279-s002.pdf]

Data Sharing Statement

Mussa. Incident Diabetes in Women With Patterns of Gestational Diabetes Occurrences Across 2 Pregnancies. *JAMA Netw Open*. Published May 09, 2024. doi:10.1001/jamanetworkopen.2024.10279

Data
Data available: No

Additional Information
Explanation for why data not available: The data that support the findings of this study are available only through Quebec's Statistical Institute Centers for Access to Research Data (CADRISQ), secure environments available to accredited researchers in Quebec for research purposes, and so are not publicly available. Restrictions apply to the availability of these data and data requests must be made with permission from the Quebec Statistical Institute (https://statistique.quebec.ca/recherche/).
